# Impact of work-family conflict on work engagement among female university teachers: Evidence from China

**DOI:** 10.1371/journal.pone.0319785

**Published:** 2025-03-25

**Authors:** Xitao Dong, Xiaona Guo, Yanyan Fu, Tingting Fu

**Affiliations:** 1 School of Economics Management, Pingdingshan University, Pingdingshan, China; 2 School of Landscape Architecture and Horticulture, Henan Forestry Vocational College, Luoyang City, China; Afghanistan Center for Epidemiological Studies, AFGHANISTAN

## Abstract

University faculty, including female teachers, often face work-family conflict (WFC) which subsequently impacts their work engagement. This study analyzes data from 489 questionnaires using Mplus 8.0 to explore the internal mechanisms linking WFC and work engagement among female faculty. The study uncovers several significant findings: (1) a negative correlation between WFC and female teachers’ work engagement; (2) job burnout mediates this relationship; (3) job crafting and grit both moderate this relationship. These insights enrich human resource management theory and offer valuable guidance for the development and implementation of university management systems.

## Introduction

Work and family roles are independent yet inherently interdependent domains [1], giving rise to phenomena such as work-family conflict (WFC) [2–4]. Due to its potential negative impact, WFC has become a hot topic of interest for scholars [5–7]. WFC, fundamentally a form of role conflict, stems from individuals’ behavioral expectations in a given position within social structures [ 8,9], reflecting work-family role incompatibility [10]. University teachers often face conflicts between teaching, research pressures, and family responsibilities. Female teachers experience even more severe WFC. On the one hand, professional women face significant role conflict challenges stemming from the necessity for increased labor force participation due to the lower pricing of the labor market in China. On the other hand, ingrained in a male-dominated culture, they shoulder greater household responsibilities, leading to dual pressures from work and family. The burden is further compounded by a greater emotional labor load, and conflicting job demands can easily lead to role conflict [8,11]. This conflict can negatively impact their mental health [9]. Many studies now confirm WFC’s negative impact on individuals’ mental and physical health [12–15] and work-related behaviors [16–19]. However, two shortcomings remain: first, there is insufficient research on the role of moderating variables in the impact process of WFC’s antecedents and consequences[20]; second, there is still a scarcity of empirical studies that incorporate individual characteristic variables into the work-family model [20].

Work engagement, proposed by Kahn [21] and defined as “the state of being cognitively, emotionally, and behaviorally connected with one’s work role”. Work engagement, a positively charged and energized state closely linked to work [22], is strongly correlated with positive work attitudes, as well as work behaviors [23] and psychological well-being [24]. It also negatively correlates with turnover rates and absenteeism [25,26]. Given work engagement’s recent introduction, research on its relationship with WFC remains limited in academia [27]. Teaching is a highly demanding profession that requires teachers to not only possess excellent subject knowledge but also significant psychological resources to cope with daily emotional challenges. Teachers are involved in considerable emotional labor in guiding, instructing, collaborating, and behaving formally and informally [28]. Job burnout is common among the teaching profession [29,30]. Job burnout involves enduring responses to long-term emotional and interpersonal stressors in the workplace [31]. Despite its detrimental effects on teachers’ professional well-being and the quality of educational services, existing interventions for teacher burnout have shown limited effectiveness [32]. Job crafting involves employees’ autonomous redefinition of job content, boundaries, and roles to foster work identification and meaning, promoting positive engagement. According to the JD-R Model, employees adjust behaviors based on abilities and needs, aiming for balance between job demands and resources [33]. Positive psychology has sparked researchers’ increased focus on individuals’ positive psychological traits and the protective factors in their development. Grit is the personality trait characterized by sustained passion and perseverance toward long-term goals [34]. It reflects the ability to maintain interest and tenacity despite significant obstacles and encompasses self-motivation, self-discipline, and self-regulation [34].

Therefore, this study empirically analyzes the relationship between WFC and work engagement among female teachers. The findings can advance human resource management theory and provide insights for school administrators on promoting teachers’ positive work behaviors.

## Research hypotheses

### WFC and work engagement

Work and family are essential aspects of individuals’ lives. In reality, individuals’ resources are limited and must be allocated wisely between work and family to achieve mutual benefit. An imbalanced allocation of these resources can lead to conflict [35], deplete individuals’ scarce resources, and bring about various negative effects, such as low job satisfaction [36], uncivil behavior [37], depression [38], role overload [39], work stress, and even organizational deviance [40]. For female teachers, balancing family and work roles is challenging. Engagement in one role often makes participation in the other more difficult [41,42]. Faced with high WFC and family role-work resource trade-offs, they boost family investment and cut work investment [43,44]. Thus, this study hypothesizes:

H1: WFC has a negative correlation with female teachers’ work engagement.

### The role of job burnout

For female teachers, they often take on caregiver roles at home, translating to increased family responsibilities. They strive to maintain a work-family balance to achieve mutual support between work and family. Role conflict creates tension among resources, resulting in burnout [45]. Therefore, the correlation between WFC and female teacher burnout is significant [46–49]. Work engagement emphasizes the positive psychological state displayed by teachers in educational contexts, including vitality, dedication, and absorption [50,51]. Job burnout, on the other hand, highlights the negative and unhealthy response resulting from prolonged work stress and resource inadequacy [51,52], leading to emotional exhaustion, reduced accomplishment, and depersonalization [52], which may adversely affect teachers’ work engagement. Thus, this study hypothesizes:

H2: Job burnout mediate the relationship between WFC and female teachers’ work engagement.

### The role of job crafting

The COR theory posits that employees reinvest resources to prevent loss and acquire more [45]. Job crafting is divided into task, relational, and cognitive crafting [53]. Job crafting can stimulate employees’ autonomy, competence, and relatedness needs, fostering intrinsic motivation, promoting organizational citizenship behavior, and enhancing work engagement [54]. Firstly, task crafting can help balance work and family relations. Female teachers can adjust the types and quantity of job tasks and pathways, integrating their actual work with personal strengths [55]. This fosters a work environment that meets their abilities and needs, leading to redesigned work boundaries and content [56]. Secondly, when individuals need to juggle work and family roles, they seek new social connections and support to avoid work engagement encroaching on the resources needed for family matters, achieving a balance between the two. Relational crafting enables employees to improve their interpersonal network by strengthening interactions with others [55], better coping with work pressure, and promoting positive work engagement behaviors. Thirdly, cognitive crafting helps employees understand the meaning of their work, significantly promoting person-job fit, work identity, and role performance [57], leading to spontaneous work engagement [55], reducing WFC, preventing burnout associated with WFC, and supporting individual well-being [58]. Thus, this study hypothesizes:

H3: Job crafting moderates the relationship between WFC and female teachers’ work engagement.

### The role of grit

The self-regulation theory suggests that positive self-regulation characteristics enable individuals to continuously adjust themselves in the pursuit of long-term goals, exert sustained effort in facing challenges, and actively seek help from various resources to achieve behavioral change [59]. As a crucial positive psychological resource for managing life stress [60], grit can help individuals demonstrate higher persistence in coping with WFC and setbacks [61], exhibit higher self-efficacy when facing work and family pressures [62], and employing emotion regulation to mitigate stress and setback impacts [63], thereby maintaining positive emotions and reducing the risk of depression [64,65]. Furthermore, individuals with high grit maintain sustained effort despite setbacks and pressures, approaching adversity with a positive mindset, leading to effective self-regulation [66]. Thus, this study hypothesizes:

H4: Grit moderates the relationship between WFC and female teachers’ work engagement.

**Fig 1:**
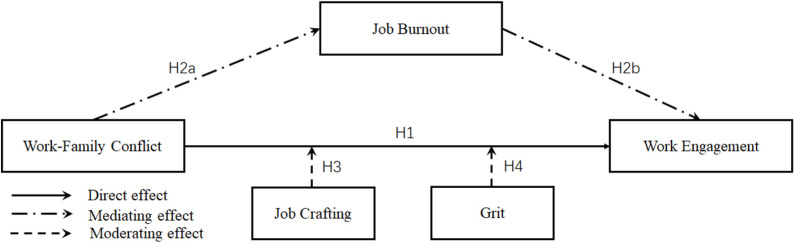
Research model.

Fig 1 illustrates the research model of this study.

## Methods

### Data collection

This study utilized a two-part questionnaire survey. The first section detailed the significance and scope of the research, providing instructions for completing the questionnaire. Additionally, an informed consent clause was included at the end of this section, allowing participants to select “Agree” to sign the consent form or “Disagree” to opt out, ensuring written informed consent. In the second part, study variable items were randomized to reduce prediction bias. The research was ethically approved by the Ethics Committee of Pingdingshan University’s School of Economics and Management prior to its initiation (Number: EA-2023039). Upon review, the study used anonymized interview data, ensured no harm came to participants, and did not involve sensitive personal information or commercial interests. The study adhered to the Declaration of Helsinki and the Belmont Report and was found to comply with the “Ethical Review Measures for Scientific and Medical Research Involving Humans” issued by the Chinese government. Furthermore, in compliance with Chinese labor laws and customary practice, this study did not include minors, as all university faculty members are adults. The study covered 56 universities, each inviting 10-12 female teachers to participate.

Data collection was conducted from May 15 to July 22, 2023. Out of 650 distributed questionnaires, 546 were returned. In this study, incomplete or logically flawed questionnaires were considered invalid. After excluding these, 489 valid responses from 52 universities were retained. The average age of respondents was 35.4 years (SD =  3.1). In terms of education, 46 (9.4%) held bachelor’s degrees, 245 (50.1%) held master’s degrees, and 198 (40.5%) held doctorate degrees. Regarding titles, 208 (42.5%) were senior, 188 (38.4%) were intermediate, and 93 (19.0%) were junior.

### Measure

WFC was measured using Netemeyer et al.‘s [10] WAFCS scale, consisting of 5 items, with a Cronbach’s α of 0.88. Work engagement was assessed using Schaufeli et al.’s [67] UWES-9 scale, including vigor, dedication, and absorption, each with three items, and a Cronbach’s α of 0.92. Job burnout was measured using Wu et al.’s [68] localized MBI-ES scale, covering emotional exhaustion, diminished achievement, and depersonalization, with a Cronbach’s α of 0.83. Job crafting was assessed using Tims & Bakker’s [69] JCS scale, encompassing three dimensions, each with five items, and a Cronbach’s α of 0.76. Grit was measured using Duckworth et al.’s [34] scale, comprising consistency of interests and perseverance of effort, totaling 12 items, with a Cronbach’s α of 0.80. All items were assessed using a seven-point Likert scale.

## Results

### Reliability and validity

Table 1 presents the results of CFA. First, since all item factor loadings for variables exceed 0.60, with CR ranging from 0.76 to 0.85, this indicates good reliability. Second, all variables have AVE values above 0.50, indicating good convergent validity. Lastly, Table 2 shows that the square roots of variables’ AVEs surpass their Pearson correlation coefficients, suggesting good discriminant validity among variables.

**Table 1:** CFA and reliability and validity tests.

Dim	Item	Estimate	S.E.	*P*	SMC	CR	AVE
**WFC**	WFC1	0.73	0.04	***	0.53	0.85	0.53
	WFC2	0.68	0.03	***	0.46		
	WFC3	0.66	0.04	***	0.44		
	WFC4	0.77	0.04	***	0.59		
	WFC5	0.79	0.03	***	0.62		
**WE**	WE1	0.76	0.02	***	0.58	0.81	0.58
	WE2	0.72	0.02	***	0.52		
	WE3	0.81	0.03	***	0.66		
**JB**	JB1	0.76	0.04	***	0.58	0.79	0.55
	JB2	0.77	0.03	***	0.59		
	JB3	0.69	0.04	***	0.48		
**JC**	JC1	0.72	0.02	***	0.52	0.76	0.51
	JC2	0.68	0.02	***	0.46		
	JC3	0.74	0.03	***	0.55		
**GT**	GT1	0.85	0.04	***	0.72	0.82	0.69
	GT2	0.81	0.03	***	0.66		

Note 1. WFC = work-family conflict; WE = work engagement; JB = job burnout; JC = job crafting; GT = grit.

**Table 2:** Test of discriminant validity.

Dim	Mean	S.D.	AVE	WFC	WE	JB	JC	GT
WFC	4.76	0.71	0.53	**0.73**				
WE	3.87	0.67	0.58	-0.48	**0.76**			
JB	4.11	0.82	0.55	0.55	-0.49	**0.74**		
JC	4.53	0.64	0.51	0.23	0.41	-0.26	**0.71**	
GT	4.47	0.66	0.69	0.21	0.46	-0.31	0.34	**0.83**

Note 2. Bold diagonal: AVE square root; lower triangle: Pearson correlation.

### Model fit

The model fit indices were *χ*^*2*^*/df* =  2.67, CFI =  0.95, TLI =  0.95, RMSEA =  0.07, and SRMR =  0.06, demonstrating that the model exhibited a good fit.

### Mediating effect

Table 3 shows a total effect of -0.37 (p <  0.001), including a direct effect of -0.26 (p <  0.001) and an indirect effect of -0.11 (p <  0.001), supporting Hypothesis 1 (H1). Additionally, a bootstrap analysis with 5,000 iterations reinforces H2.

**Table 3:** Test of mediating effect.

	Estimate	Coefficients			95% CI		Hypothesis
		SE	Est./SE	P-Value	Lower 2.5%	Upper 2.5%	(Y/N)
**Total**	-0.37	0.05	7.89	***	-0.48	-0.27	
**Dir**	-0.26	0.04	6.85	***	-0.34	-0.18	H1(Y)
**Ind**	-0.11	0.03	3.73	***	-0.18	-0.05	H2(Y)

### Moderating effect

**Fig 2:**
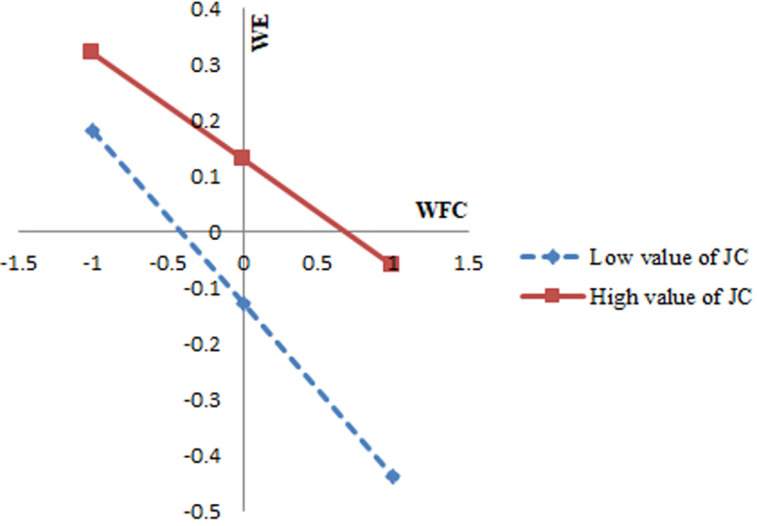
The role of job crafting (JC).

**Fig 3:**
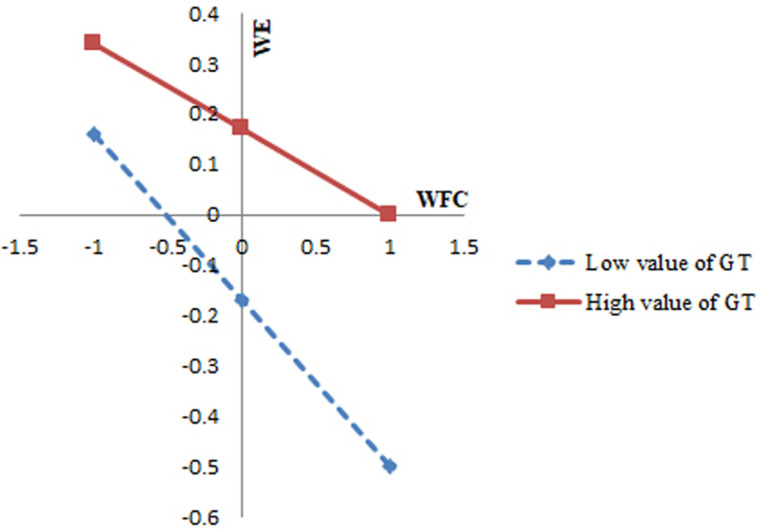
The role of grit (GT).

Table 4 shows that the interaction effect between WFC and job crafting is 0.06 (p <  0.01), supporting Hypothesis 3 (H3). Similarly, the interaction effect between WFC and grit is 0.08 (p <  0.01), confirming Hypothesis 4 (H4). Fig 2 illustrates the moderating effect of job crafting. As job crafting decreases, the slope becomes steeper, indicating that lower job crafting results in a stronger negative impact of WFC on female teachers’ work involvement. Fig 3 displays the moderating effect of grit. A decrease in grit leads to a steeper slope, suggesting that lower grit exacerbates the negative impact of WFC on female teachers’ work engagement.

**Table 4:** Test of moderating effect.

DV	IV	Estimate	SE	Est./SE	P-Value	Hypothesis (Y/N)
**WE**	**WFC**	-0.25	0.04	-6.95	***	
	**JB**	0.16	0.03	9.94	***	
	**JC**	0.13	0.03	6.04	***	
	**GT**	0.17	0.03	-7.06	***	
	WFC*JC	0.06	0.02	-3.04	**	H3 (Y)
	WFC*GT	0.08	0.02	-3.46	***	H4 (Y)

## Discussion

### Theoretical contributions

Previous studies on the antecedents and consequences of WFC have mainly focused on mediating variables [16], often overlooking the impact of moderating variables [20]. This research aims to bridge this gap by constructing a moderated mediation model to explore the underlying mechanisms between WFC and work behaviors. Furthermore, existing research has primarily emphasized organizational-level variables [8,70,71]. This study complements these findings by integrating individual-level variables such as job crafting and grit, thereby enriching the understanding of this domain.

### Practical implications

First, mitigate WFC by clearly delineating boundaries between work and family life, including specific time and spaces for each [72]. Sharing family responsibilities with relatives, friends, and family members can help lighten the burdens of both work and family tasks. Seeking support and utilizing trustworthy childcare and domestic services can alleviate the pressure of family responsibilities.

Second, combat work fatigue by setting clear work goals and developing feasible plans and schedules to foster a sense of purpose and achievement. Fostering positive communication and collaboration with colleagues and superiors, and participating in training and professional organizations, enhances job satisfaction and skills.

Third, enhance work-shaping capabilities by defining career development goals and translating them into specific action plans. Actively participating in training and workshops facilitates ongoing improvement, while positive relationships with colleagues and partners create more opportunities for career growth.

Lastly, foster the virtue of perseverance by adopting a positive mindset to cultivate patience and resilience when facing challenges. Embracing challenges as opportunities for personal growth, seeking solutions, and adapting to changes actively contribute to regaining strength during setbacks [73]. Developing self-discipline habits such as effective time management, a balanced diet, and sufficient sleep nurtures patience and resilience.

### Limitations and future directions

On one hand, our study employs cross-sectional data. Although the relevant data have undergone CFA and model fitness testing, cross-sectional data inherently have limitations in inferring causal relationships between variables. Future research could adopt longitudinal data to more accurately deduce causal relationships among variables. On the other hand, the current study focused solely on individual-level variables. In the future, a multi-level research approach could be utilized to further explore the impact of organizational-level variables. This would provide a more comprehensive understanding of the factors influencing the outcomes.

## Conclusions

This study finds a negative correlation between WFC and engagement, with job burnout mediating this relationship and both job crafting and grit moderating it. These findings significantly enrich the theoretical framework of HRM and offer practical guidance for the development and implementation of university management systems. By understanding and addressing the interplay between WFC, job burnout, job crafting, and grit, institutions can foster a more supportive environment that not only enhances the work engagement but also promotes the overall well-being of their female faculty members.

## Supporting information

S1 Questionnaire Original questionnaire (1004). (PDF)
