# Supplementary material for: Impact of work-family conflict on work engagement among female university teachers: Evidence from China
Source: PLoS One. 2025 Mar 25;20(3):e0319785. doi: 10.1371/journal.pone.0319785 (PMC11936228; doi:10.1371/journal.pone.0319785)
Supplement: S1 Questionnaire — (PDF) [file pone.0319785.s001.pdf]

# Original Questionnaire

Dear Madam,

We are planning to conduct a study on work-family conflict and work engagement among female teachers in higher education. This research can not only contribute to theoretical advancements in the field of human resource management but also provide valuable insights for school administrators on how to effectively promote positive work behaviors among teachers. Therefore, we sincerely appreciate your participation in this survey. We assure you that the survey will be conducted anonymously, and the data collected will be used solely for scientific research purposes, never for any commercial applications. We are grateful for your generous contribution.

If you agree to the above terms, please select the "Agree" option below (***Note: Selecting "Agree" indicates that you have signed the informed consent form***); if you do not agree to the above terms, please select the "Disagree" option, in which case you may decline or withdraw from completing the survey.

**Agree:** **Disagree:**

## Basic Information:

1. Your institution is located in:
   Eastern China ( ) Central China ( ) Western China ( )
2. Age:
3. Education Level: Bachelor's degree and below ( ) Master ( ) Doctorate ( )
4. Title: Senior titles ( ) Intermediate titles ( ) Junior titles ( )

## Items:

Please use a scale from 1 to 7 to indicate your level of agreement with the statements below. A rating of "7" means you strongly agree, while a rating of "1" means you strongly disagree.

***Note: In the original questionnaire, to prevent participants from guessing the item information, the order of the following items was randomized. Here, for clarity and convenience for the reader to understand how each variable is measured, we have restored the order of the items according to their respective scales.***

**Items measuring work-family conflict**

1. My work interferes with my family and home life.

1 ( ) 2 ( ) 3 ( ) 4 ( ) 5 ( ) 6 ( ) 7 ( )

2. The long hours of my work make it difficult to fulfill family responsibilities.

1 ( ) 2 ( ) 3 ( ) 4 ( ) 5 ( ) 6 ( ) 7 ( )

3. Things I want to do at home remain undone because of the demands of my job.

1 ( ) 2 ( ) 3 ( ) 4 ( ) 5 ( ) 6 ( ) 7 ( )

4. The high stress of my job makes it difficult to fulfill family responsibilities.

1 ( ) 2 ( ) 3 ( ) 4 ( ) 5 ( ) 6 ( ) 7 ( )

5. I have had to change my family's activity plans due to work reasons.

1 ( ) 2 ( ) 3 ( ) 4 ( ) 5 ( ) 6 ( ) 7 ( )

**Items measuring work engagement**

1. I am enthusiastic about my work.

1 ( ) 2 ( ) 3 ( ) 4 ( ) 5 ( ) 6 ( ) 7 ( )

2. I feel actively engaged in my work.

1 ( ) 2 ( ) 3 ( ) 4 ( ) 5 ( ) 6 ( ) 7 ( )

3. I have a lot of energy for my job.

1 ( ) 2 ( ) 3 ( ) 4 ( ) 5 ( ) 6 ( ) 7 ( )

4. I am strongly committed to my work.

1 ( ) 2 ( ) 3 ( ) 4 ( ) 5 ( ) 6 ( ) 7 ( )

5. I am very focused on my work.

1 ( ) 2 ( ) 3 ( ) 4 ( ) 5 ( ) 6 ( ) 7 ( )

6. I put a lot of effort into my work.

1 ( ) 2 ( ) 3 ( ) 4 ( ) 5 ( ) 6 ( ) 7 ( )

7. I feel I have the opportunity to use my skills in my job.

1 ( ) 2 ( ) 3 ( ) 4 ( ) 5 ( ) 6 ( ) 7 ( )

8. I feel highly involved in my work.

1 ( ) 2 ( ) 3 ( ) 4 ( ) 5 ( ) 6 ( ) 7 ( )

9. I am proud of my work.

1 ( ) 2 ( ) 3 ( ) 4 ( ) 5 ( ) 6 ( ) 7 ( )

**Items measuring job burnout**

1. I feel like I'm overdrawing on my life.

1 ( ) 2 ( ) 3 ( ) 4 ( ) 5 ( ) 6 ( ) 7 ( )

2. I find teaching to be an exhausting job.

1 ( ) 2 ( ) 3 ( ) 4 ( ) 5 ( ) 6 ( ) 7 ( )

3. After a day's work, I feel utterly drained.

1 ( ) 2 ( ) 3 ( ) 4 ( ) 5 ( ) 6 ( ) 7 ( )

4. I feel that teaching depletes my emotions and energy.

1 ( ) 2 ( ) 3 ( ) 4 ( ) 5 ( ) 6 ( ) 7 ( )

5. I feel a sense of being drained in my work.

1 ( ) 2 ( ) 3 ( ) 4 ( ) 5 ( ) 6 ( ) 7 ( )

6. I find it easy to create a relaxed atmosphere when I'm with students.

1 ( ) 2 ( ) 3 ( ) 4 ( ) 5 ( ) 6 ( ) 7 ( )

7. I have accomplished valuable things in my work.

1 ( ) 2 ( ) 3 ( ) 4 ( ) 5 ( ) 6 ( ) 7 ( )

8. I am able to help students find confidence.

1 ( ) 2 ( ) 3 ( ) 4 ( ) 5 ( ) 6 ( ) 7 ( )

9. I can effectively handle students' problems.

1 ( ) 2 ( ) 3 ( ) 4 ( ) 5 ( ) 6 ( ) 7 ( )

10. I can provide students with beneficial guidance.

1 ( ) 2 ( ) 3 ( ) 4 ( ) 5 ( ) 6 ( ) 7 ( )

11. I find myself scolding students harshly over trivial matters.

1 ( ) 2 ( ) 3 ( ) 4 ( ) 5 ( ) 6 ( ) 7 ( )

12. I am very demanding of students.

1 ( ) 2 ( ) 3 ( ) 4 ( ) 5 ( ) 6 ( ) 7 ( )

13. I have the urge to scold students.

1 ( ) 2 ( ) 3 ( ) 4 ( ) 5 ( ) 6 ( ) 7 ( )

14. I feel that students are dissatisfied with my way of handling problems.

1 (  )  2 (  )  3 (  )  4 (  )  5 (  )  6 (  )  7 (  )

15. I feel like I often treat students as lifeless objects.

1 (  )  2 (  )  3 (  )  4 (  )  5 (  )  6 (  )  7 (  )

**Items measuring job crafting**

1. I will adopt new methods to improve my work.

1 (  )  2 (  )  3 (  )  4 (  )  5 (  )  6 (  )  7 (  )

2. I will proactively adjust the scope or type of tasks to complete my work.

1 (  )  2 (  )  3 (  )  4 (  )  5 (  )  6 (  )  7 (  )

3. I tend to choose jobs that align with my skills when making job choices.

1 (  )  2 (  )  3 (  )  4 (  )  5 (  )  6 (  )  7 (  )

4. I am willing to take on additional work tasks in my job.

1 (  )  2 (  )  3 (  )  4 (  )  5 (  )  6 (  )  7 (  )

5. When choosing a job, I prioritize jobs that I am interested in.

1 (  )  2 (  )  3 (  )  4 (  )  5 (  )  6 (  )  7 (  )

6. The attainment of my own work goals gives my life a sense of purpose.

1 (  )  2 (  )  3 (  )  4 (  )  5 (  )  6 (  )  7 (  )

7. My work is highly important for achieving organizational goals.

1 (  )  2 (  )  3 (  )  4 (  )  5 (  )  6 (  )  7 (  )

8. My work contributes significantly to societal progress.

1 (  )  2 (  )  3 (  )  4 (  )  5 (  )  6 (  )  7 (  )

9. I believe that my work has a positive impact on my life.

1 (  )  2 (  )  3 (  )  4 (  )  5 (  )  6 (  )  7 (  )

10. I believe that work contributes to my overall perception of happiness.

1 (  )  2 (  )  3 (  )  4 (  )  5 (  )  6 (  )  7 (  )

11. In my work, I make an effort to understand others.

1 (  )  2 (  )  3 (  )  4 (  )  5 (  )  6 (  )  7 (  )

12. I actively participate in work-related group activities.

1 (  )  2 (  )  3 (  )  4 (  )  5 (  )  6 (  )  7 (  )

13. I organize informal activities within my team/organization.

1 ( ) 2 ( ) 3 ( ) 4 ( ) 5 ( ) 6 ( ) 7 ( )

14. I provide assistance to new colleagues for unfamiliar issues.

1 ( ) 2 ( ) 3 ( ) 4 ( ) 5 ( ) 6 ( ) 7 ( )

15. I make friends with people in the organization who share similar interests or skills as me.

1 ( ) 2 ( ) 3 ( ) 4 ( ) 5 ( ) 6 ( ) 7 ( )

**Items measuring grit**

1. I often set a goal but later choose to pursue a different one.

1 ( ) 2 ( ) 3 ( ) 4 ( ) 5 ( ) 6 ( ) 7 ( )

2. New ideas and new projects sometimes distract me from previous ones.

1 ( ) 2 ( ) 3 ( ) 4 ( ) 5 ( ) 6 ( ) 7 ( )

3. I become interested in new pursuits every few months.

1 ( ) 2 ( ) 3 ( ) 4 ( ) 5 ( ) 6 ( ) 7 ( )

4. My interests change from year to year.

1 ( ) 2 ( ) 3 ( ) 4 ( ) 5 ( ) 6 ( ) 7 ( )

5. I have been obsessed with a certain idea or project for a short time but later lost interest.

1 ( ) 2 ( ) 3 ( ) 4 ( ) 5 ( ) 6 ( ) 7 ( )

6. I have difficulty maintaining my focus on projects that take more than a few months to complete.

1 ( ) 2 ( ) 3 ( ) 4 ( ) 5 ( ) 6 ( ) 7 ( )

7. I have achieved a goal that took years of work.

1 ( ) 2 ( ) 3 ( ) 4 ( ) 5 ( ) 6 ( ) 7 ( )

8. I have overcome setbacks to conquer an important challenge.

1 ( ) 2 ( ) 3 ( ) 4 ( ) 5 ( ) 6 ( ) 7 ( )

9. I finish whatever I begin.

1 ( ) 2 ( ) 3 ( ) 4 ( ) 5 ( ) 6 ( ) 7 ( )

10. Setbacks don’t discourage me.

1 ( ) 2 ( ) 3 ( ) 4 ( ) 5 ( ) 6 ( ) 7 ( )

11. I am a hard worker.

1 ( ) 2 ( ) 3 ( ) 4 ( ) 5 ( ) 6 ( ) 7 ( )

12. I am diligent. 1 ( ) 2 ( ) 3 ( ) 4 ( ) 5 ( ) 6 ( ) 7 ( )
